# Dab2IP GTPase Activating Protein Regulates Dendrite Development and Synapse Number in Cerebellum

**DOI:** 10.1371/journal.pone.0053635

**Published:** 2013-01-09

**Authors:** Shuhong Qiao, Sun-Hong Kim, Detlef Heck, Daniel Goldowitz, Mark S. LeDoux, Ramin Homayouni

**Affiliations:** 1 Department of Biological Sciences, University of Memphis, Memphis, Tennessee, United States of America; 2 Department of Anatomy and Neurobiology, University of Tennessee Health Science Center, Memphis, Tennessee, United States of America; 3 Department of Neurology, University of Tennessee Health Science Center, Memphis, Tennessee, United States of America; University of Nebraska Medical Center, United States of America

## Abstract

DOC-2/DAB-2 interacting protein (Dab2IP) is a GTPase activating protein that binds to Disabled-1, a cytosolic adapter protein involved in Reelin signaling and brain development. Dab2IP regulates PI3K-AKT signaling and is associated with metastatic prostate cancer, abdominal aortic aneurysms and coronary heart disease. To date, the physiological function of Dab2IP in the nervous system, where it is highly expressed, is relatively unknown. In this study, we generated a mouse model with a targeted disruption of *Dab2IP* using a retrovirus gene trap strategy. Unlike *reeler* mice, Dab2IP knock-down mice did not exhibit severe ataxia or cerebellar hypoplasia. However, Dab2IP deficiency produced a number of cerebellar abnormalities such as a delay in the development of Purkinje cell (PC) dendrites, a decrease in the parallel fiber synaptic marker VGluT1, and an increase in the climbing fiber synaptic marker VGluT2. These findings demonstrate for the first time that Dab2IP plays an important role in dendrite development and regulates the number of synapses in the cerebellum.

## Introduction

Reelin signaling pathway controls neuronal migration, dendrite maturation, and synaptic plasticity [1]–[4]. Reelin is a large extracellular glycoprotein that binds to lipoprotein receptors ApoER2 and VLDLR, resulting in tyrosine phosphorylation of cytosolic adapter protein Disabled-1 (Dab1) by src family tyrosine kinases [5]–[8]. Tyrosine phosphorylated Dab1 binds to the p85 regulatory subunit of PI3K, and CrkL, an upstream activator of Rap1 signaling [9]–[11]. Disruption of Reelin signaling in mice results in a distinct ataxic behavior, severe hypoplasia of the cerebellum, and abnormal lamination of cortical structures [12]–[17]. To identify other molecules in the Reelin signaling pathway, we previously searched for Dab1 interacting proteins using a yeast two hybrid approach. We found that the PTB domain of Dab1 specifically interacted with the NPxY motif in Disabled homolog 2 interacting protein (Dab2IP) [18]. Recently, we showed that *in utero* knock-down of Dab2IP in mice disrupts migration of late-born cortical neurons [19].

Dab2IP is a member of the Ras GTPase-Activating Protein (GAP) which was identified independently several years ago via its interaction with disabled-2 (Dab2), disabled-1 (Dab1), and apoptosis signal regulating kinase 1 (ASK1) [18], [20]–[22]. Dab2IP functions as a tumor suppressor protein, regulating epithelial-to-mesenchymal transition and prostate cancer metastasis [21], [23], [24]. It modulates the balance between phosphatidylinositol 3-kinase (PI3K) mediated cell survival and ASK1 mediated apoptosis [25], [26]. Dab2IP causes dissociation of ASK1 from its inhibitor and activation of c-Jun Kinase (JNK) signaling, while at the same time activating IKK NF-kB signaling via TRAF2 [22], [25]. In addition, the proline-rich region of Dab2IP was shown to bind the p85 regulatory subunit of PI3K, resulting in inhibition of PI3K-AKT signaling and suppression of cell survival and proliferation [26]. Recently, sequence variants in *DAB2IP* have been linked to aggressive metastatic prostate cancer [27], abdominal aortic aneurysms [28], and coronary heart disease [29].

To investigate the role of Dab2IP in brain and in Reelin signaling, we generated a mouse model in which *Dab2IP* gene was disrupted by a retroviral gene trap strategy. This strategy resulted in a partial knock-out of Dab2IP, deleting 2 of the 3 major protein isoforms in the brain. Dab2IP knock-down (KD) mice were viable, fertile and did not exhibit the classical *reeler*-like ataxia. However, we found that Dab2IP plays an important role in development of Purkinje cell dendrites and formation of cerebellar synaptic structures.

## Materials and Methods

### Ethics Statement

All mice used in this study were maintained in certified animal facilities either at the University of Tennessee Health Science Center or at the University of Memphis. All experiments were performed in accord with the institutional guide for animal care using an animal protocol (protocol #0644, April 11, 2008) approved by the University of Memphis Institutional Animal Care and Use Committee (IACUC). Throughout all experimental procedures, efforts were made to minimize the numbers of the animals used and their suffering.

### Generation of Dab2IP KD Mice

Targeted embryonic stem (ES) cells (Omni Bank® Clone OST348452) were obtained from Lexicon (The Woodlands, TX, USA) which had a retroviral gene-trap cassette insertion into the *Dab2IP* gene locus. The gene-trap cassette contains a β-geo reporter gene (a fusion gene of β-galactosidase and neomycin phosphotransferase II) as described previously by Zambrowicz and colleagues [30]. Chimeric mice were generated by injection of the 129 strain-derived ES cells into C57BL/6 blastocysts at the University of Tennessee Health Science Center Transgenic Core Facility. Male chimeric mice were mated with wild type C57BL/6 females to obtain germline transmission of the *Dab2IP* transgene, resulting in production of the *Dab2IP* KD (*Dab2IP* ^Gt(OST348452)Lex^) mice. The presence of the transgene was detected by PCR using the following primers: wild-type forward primer (5′-TGGACCGCAACCACAGCTTCCGC-3′), wild-type reverse primer (5′-CCTACCTCTAGGCACAGCACTGC-3′), and β-geo forward primer (5′-TGGCGTTACTTAAGCTAGCTTGC-3′).

### Immunoblot Analysis

Cerebella were excised from P30 anesthetized mice and homogenized in lysis buffer [50 mM Tris-HCl (pH 7.4), 150 mM NaCl, 1% NP-40, 10% glycerol, 1 mM PMSF, 10 µg/ml aprotinin, 10 µg/ml leupeptin]. Protein concentration was determined using the BCA protein assay (Pierce). Thirty µg/lane of each homogenate were separated by SDS-PAGE using 7.5% Criterion Tris-Hcl gels (Bio-Rad) and transferred to nitrocellulose membrane (Whatman). Blots were blocked in 5% nonfat milk in 1×TBST for 1.5 h at room temperature followed by incubated with primary antibodies (1∶5,000 in 5% milk/1×TBST) at 4°C overnight, and then secondary antibodies (1∶10,000 in 5% milk/1×TBST) 1 h at room temperature. After washing, the proteins were visualized using Supersignal West Pico chemiluminescent detection system (Pierce).

### Quantitative Real-time RT-PCR

Total RNA was isolated form P30 wild-type (WT) and Dab2IP KD littermate cerebella using TRIzol reagent (Invitrogen). Quantitative RT-PCR was performed on a LightCycler 480 Real-Time PCR System (Roche) using the following probes according to manufacturer’s protocols. The following forward and reverse primers were used: GRD domain, 5′-GCC TTC TGC AAG ATC ATC AAC (forward) and 5′-GCT GAT GAG CCG TTC ACT G (reverse); PH domain, 5′-CGC GGA CAA TGA GAG GTC (forward) and 5′-GAG CAG GGA CTC GTG TGA C (reverse). RT-PCR reactions were performed as follows: initial denaturation at 95°C for 5 minutes, followed by 50 cycles of 95°C for 10 s, 60°C for 30 s, and 72°C for 10 s, and final cooling at 40°C for 10 seconds. The product sizes were confirmed by agarose gel electrophoresis, and melting curves were analyzed to control the specificity of PCR reactions. Dab2IP expression levels were normalized to β-actin, 40S ribosomal protein, and S19 levels. The relative levels of Dab2IP expression were measured by a modified ΔΔCt [31].

### Histology and Immunohistochemistry

Mice were deeply anesthetized (avertin), perfused through the aorta with ice-cold 4% paraformaldehyde, and equilibrate with 30% sucrose overnight. Brains were embedded in tissue freezing medium (OCT), frozen in isopentane cooled with liquid nitrogen, and sectioned using a Leica cryostat (CM3050). Parasagittal sections (10 µm or 6 µm) were examined by Nissl or immunohistochemical staining. For immunohistochemical analysis, sections were incubated with anti-calbindin (1∶1000, mosue, Abcam) or anti-Dab2IP (1∶3000, rabbit) antibodies [18], followed by blocking with PBS containing 1% bovine serum albumin and 4% normal horse serum. Immunoreactivity was visualized by using ABC kit (Vector) and diaminobenzidine (DAB, Vector). For immunofluoresence analysis, sagittal sections were incubated with blocking solution (10% normal serum in 0.25% Triton X-100 in PBS) for 1 h at room temperature and then incubated with the following antibodies overnight at 4°C: Rabbit anti-Dab2IP [18], anti-Calbindin (1∶500, mouse, Abcam), anti-GFAP (1∶800, mouse, Chemicon), anti-NeuN (1∶800, mouse, Millipore), anti-parvalbumin (1∶500, mouse, Millipore), anti-Vesicular Glutamate Transporter Type 1 (VGluT1,1∶500, mouse, Millipore), anti-Vesicular Glutamate Transporter Type 2 (VGluT2, guinea-pig, 1∶2000, Millipore), anti-GluRdelta2 (1∶1000, goat, Santa Cruz). After washing, sections were incubated with Alexa Fluor (Invitrogen) conjugated secondary antibodies (1∶2000) for 2 h at room temperature and rinsed in PBS. Sections were mounted with anti-fading agent (Invitrogen) and examined with LSM 710 Zeiss confocal laser scanning microscope (Zeiss, Germany).

### Quantitative Analysis

Immunofluorescence images were obtained using an LSM 710 Zeiss confocal laser-scanning microscope (Zeiss, Germany). For evaluation of the PF terminals in the cerebellum, mid-sagittal sections (6 µm each) from wild-type (N = 3) and Dab2IP KD (N = 3) littermates were double-labeled with anti-VGluT1 and anti-Calbindin. For each pair of littermates, 10 plate-matched sections were analyzed. For each section, three separate images were collected from the most distal part of the molecular layer within lobule IV/V as shown in Figure S1. Images were obtained using a 63×oil-immersion objective with a zoom factor of 3 (resolution of 1,024×1,024). A single scanned confocal plane was split into 4 quadrants and the number of puncta was counted in a blinded manner as demonstrated in Figure S1. The number of VGluT1-positive varicosities per 100 µm^2^ was averaged across multiple images and sections for each WT and Dab2IP KD animal and a Student’s *t* test was performed to determine statistical significance.

For the evaluation of the CF terminations on proximal PC dendrites, sagittal sections (6 µm) of cerebella were double labeled with anti-VGluT2 and anti-Calbindin antibodies. Images were taken on Lobule IV/V using 40×oil immersion objective. VGluT2 positive varicosities were manually counted within 200 µm wide columns divided in five equal segments along the entire length of PC dendrites. The number of VGluT2 positive varicosities was averaged from three independent KD and WT mice. Statistical analysis was conducted using the Student’s *t* test.

## Results and Discussion

### Dab2IP Expression in Cerebellum

**Figure 1:**
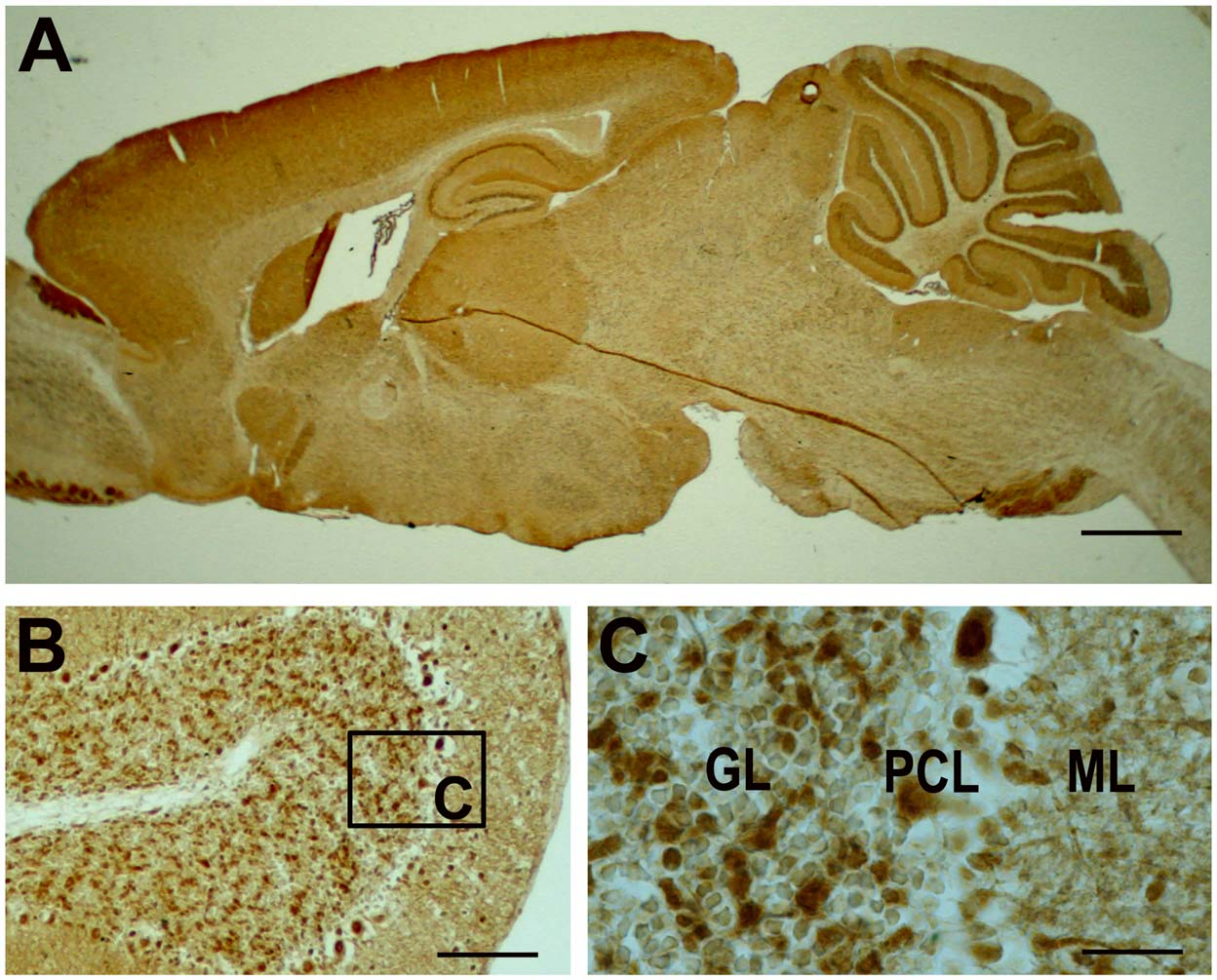
Dab2IP expression in brain. (A) Immunohistochemical staining of sagittal brain section of P30 mouse using rabbit polyclonal antiserum specific to Dab2IP. Dab2IP is highly expressed throughout the brain. The distance of the sections from the midline of the cerebellum is ∼0.4 mm. (B) In the cerebellum, Dab2IP is expressed in granule cell layer, Purkinje cells bodies and dendrites and molecular layer. (C) Higher magnification of boxed area in B. GL, granule cell layer. ML, molecular layer. Scale bars: 250 µm (A), 100 µm (B), 25 µm (C).

**Figure 2:**
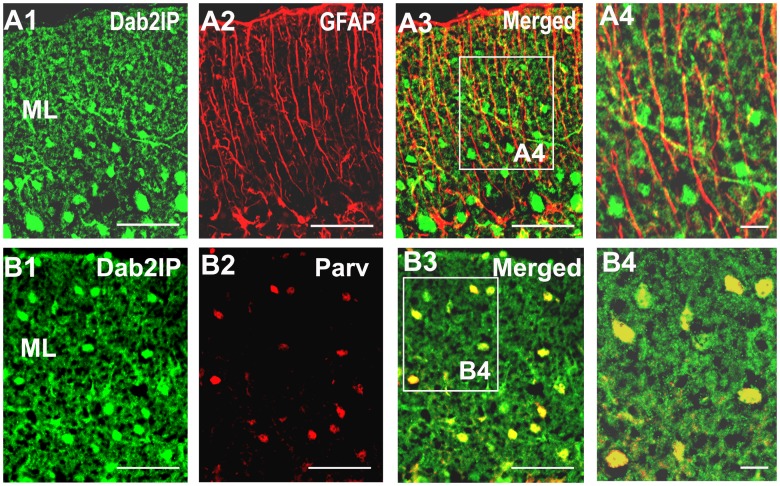
Cellular distribution of Dab2IP in the molecular layer of P30 cerebellum. (A1–A4) Double immunofluorescent labeling of Dab2IP (green) and GFAP (red) in sagittal sections of P30 mouse cerebellum. (A4) Higher magnification of boxed region in A3. (B1–B4) Double fluorescent labeling of Dab2IP (green) and Parvalbumin (red) in sagittal sections of P30 mouse cerebellum. (B4) Higher magnification of boxed region in B3. C4 is a higher magnification of the boxed area in C3. GL, granule cell layer, PCL, Purkinje cell layer; ML, molecular layer. Scale bars: 50 µm (A1–A3, B1–B3), 10 µm (A4, B4).

To determine the expression pattern of Dab2IP, we performed immunohistochemical analysis using Dab2IP specific antisera on mid-sagittal brain sections of P30 wild-type (WT) mice. Dab2IP was widely expressed throughout the brain, including olfactory bulb, hypothalamus, cerebellum and cerebral cortex (Fig. 1A). In the cerebellum, Dab2IP immunoreactive puncta were distributed at moderate intensities in the molecular layer (Fig. 1C) and dense Dab2IP staining was observed in glomerular-like structures in the granule cell layer (Fig. 1B, 1C). In addition, moderate to high Dab2IP staining was observed in soma and dendrites of Purkinje cells (Fig. 1C) as well as scattered cell bodies in the molecular layer (Fig. 1C; Fig. 2B1) of the cerebellum.

To determine which cell types express Dab2IP in the cerebellum, double immunofluorescence staining was performed using anti-Dab2IP antibody along with various antibodies against neuronal and glial marker proteins (Fig. 2). We found that Dab2IP expression was clearly absent in GFAP-positive Bergman glial fibers (Fig. 2A1–A4). However, Dab2IP was highly expressed in parvalbumin-positive interneurons in the molecular layer (Fig. 2B1–B4). These results suggest that Dab2IP is specifically expressed in neurons in the cerebellum.

**Figure 3:**
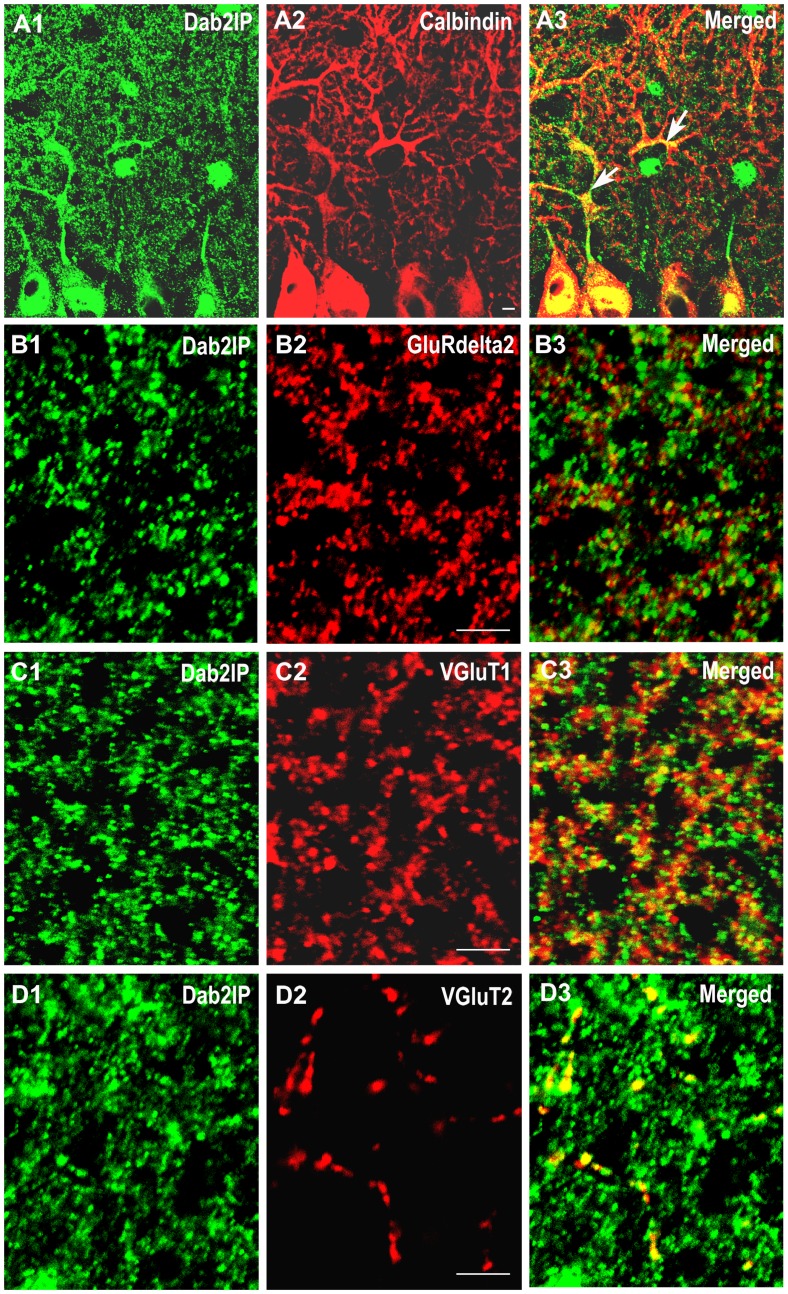
Cellular distribution of Dab2IP in P30 cerebellar Purkinje cells and granule cell layer. (A1–A3) Double immunofluorescent labeling of Dab2IP (green) and Calbindin (red) in Purkinje cell layer. (B1–B3) Double immunofluorescent labeling of Dab2IP (green) and GluR delta2 (red) in the molecular layer of the cerebellum. (C1–C3) Double immunofluorescent labeling of Dab2IP (green) and VGluT1 (red) in the granular layer of the cerebellum. (D1–D3) Double immunofluorescent labeling of Dab2IP (green) and VGluT2 (red) in the granular layer of the cerebellum. Scale bars: 5 µm.

To determine the precise cellular and sub-cellular distribution of Dab2IP, double immunofluorescence staining was performed using antibodies against Dab2IP along with either calbindin or different pre- or post-synaptic markers (Fig. 3). Dab2IP was expressed in calbindin-positive Purkinje cell bodies and dendrites along with punctate staining throughout the molecular layer (Fig. 3A). Interestingly, Dab2IP did not co-localize with glutamate receptor delta 2 (GluRδ2), a member of the ionotropic glutamate receptor (iGluR) family which is predominantly expressed in the postsynaptic densities in PC dendrites (Fig. 3B) [32]–[34]. These results indicate that Dab2IP is expressed in PC dendrites but not in post-synaptic densities in PCs. Purkinje cell dendrites form synapses with excitatory parallel fibers (PFs) in distal regions, whereas climbing fibers (CFs) form synaptic contacts at proximal regions of PC dendrites in the molecular layer. We detected strong co-localization of Dab2IP with both VGluT1 and VGluT2 (Fig. 3C and 3D). These results indicate that Dab2IP is expressed in presynaptic varicosities in the molecular layer of the cerebellum.

**Figure 4:**
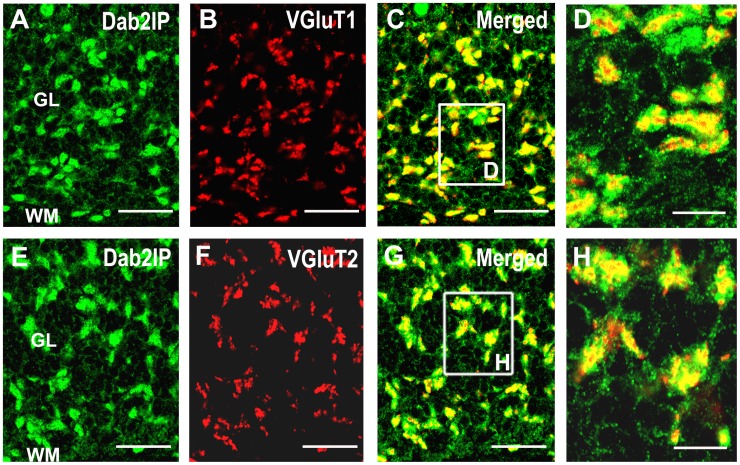
Dab2IP is expressed in mossy fiber terminals in the granule cell layer. (A–C) Double immunofluorescent labeling of Dab2IP (green) and VGluT1 (red) in cerebellar granule layer of WT P30 mice. (D) Higher magnification of the boxed area in C. (E–G) Double immunofluorescent labeling of Dab2IP (green) and VGluT2 (red) in cerebellar granule layer of WT P30 mice. (H) Higher magnification of the boxed area in G. GL, granule cell layer. Scale bars: 50 µm (A–C, E–G), 10 µm (D and H).

Granule cells in the cerebellum receive excitatory input from mossy fiber terminals localized in glomerular structures in the granule cell layer. Three types of mossy fibers have been identified based on immunoreactivity for VGluT1 and/or VGluT2 [35]. We found that Dab2IP co-localizes with both VGluT1 and VGluT2 positive mossy fiber terminals in the granule cell layer of the cerebellum (Fig. 4).

Taken together, these results indicate that Dab2IP is expressed in various cerebellar neurons and is distributed in soma, dendrites and axons. In addition, Dab2IP appears to be localized to presynaptic structures (parallel fibers, climbing fibers and mossy fibers) but is absent in post-synaptic structures of PCs, in spite of being highly expressed in PC dendrites. Thus, Dab2IP may have multiple molecular functions associated with its precise cellular and subcellular distributions.

### Generation and Characterization of Dab2IP Knock-down Mice

**Figure 5:**
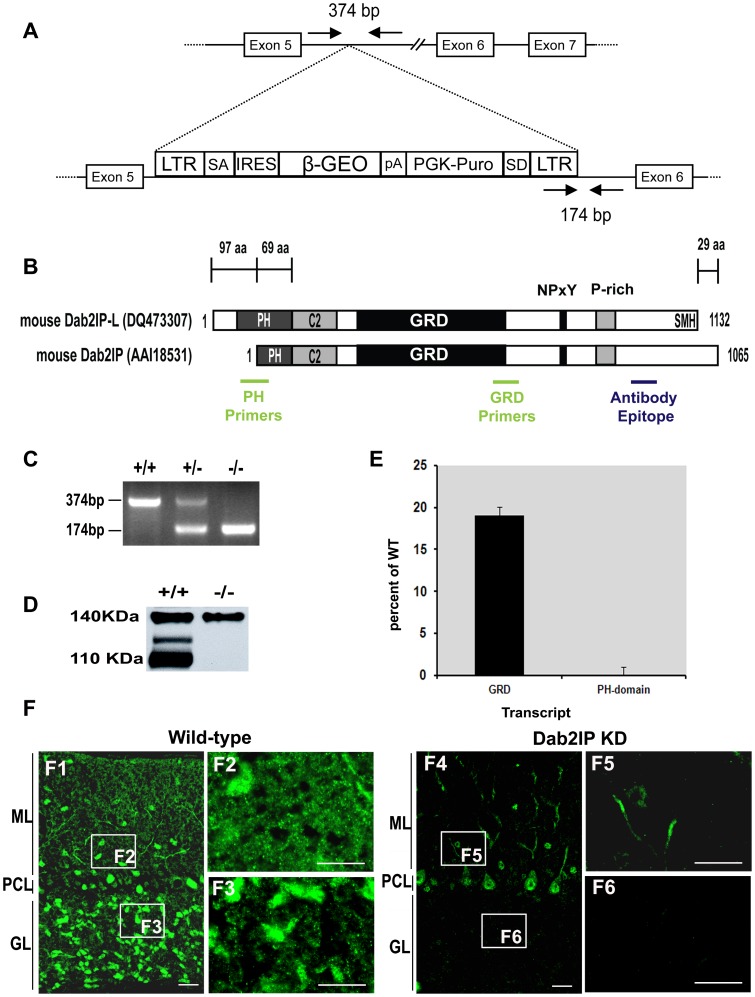
Dab2IP KD strategy and validation. (A) Structure of the gene trap cassette and its insertion site in *Dab2IP*. (B) Domain structure of two Dab2IP isoforms found in mouse. The location of PCR primers specifically targeting the PH or GRD domains are indicated in green. The location of the epitope for the polyclonal anti-Dab2IP antisera used in this study is indicated in blue. (C) PCR genotyping assay for detection of the gene trap cassette (−/−) or WT locus (+/+). The location of the primers is indicated in A. (D) Immunoblot analysis of brain lysates from P30 WT (+/+) and Dab2IP KD (−/−) mice. (E) Quantitative RT-PCR using probes to either the GRD domain (common to all isoforms) or PH-domain. Values are presented as percent change in Dab2IP KD compared to WT controls. (F) Immunodetection of Dab2IP in sagittal cerebellar sections of P30 WT (F1–F3) and Dab2IP KD (F4–F6) littermates. ML, molecular layer; PCL, Purkinje cell layer; GL, granule layer. Scale bars, 50 µm (F1, F4); 20 µm (F2, F3, F5, F6).

To investigate the function of Dab2IP in brain, we generated mice which contained a targeted disruption of *Dab2IP* using a retroviral gene trap strategy. Sequence analysis and PCR assays revealed that the retroviral cassette was inserted between exons 5 and 6 of *Dab2IP* (Fig. 5A). Since the identification of rat DOC-2/DAB-2 interacting protein (DIP1/2) [20], [21], several *Dab2IP* transcripts have been isolated from human and rodents. In rodents, at least two *Dab2IP* transcripts have been reported (Fig. 5B). Previously, we isolated a partial Dab2IP cDNA from mouse brain which displayed high sequence identity to rat DIP1/2 [18]. Using the EST and mouse genome sequence information at UCSC Genome Browser (http://genome.ucsc.edu/), we cloned a longer transcript variant of mouse Dab2IP from adult mouse brain by RT-PCR (GenBank accession no. DQ473307; Fig. 5B) and named it Dab2IP-L (Dab2IP long form). Dab2IP-L transcript encodes a longer pleckstrin homology (PH) domain, which contains an additional N terminal 97 amino acid residues compared to other reported transcripts. While there is variability in the 5′-region of Dab2IP transcripts, all transcripts seem to share a core central region which contain a PKC conserved 2 (C2) domain, a GAP-related domain (GRD), an NPXY motif, and a proline-rich region (Fig. 5B).

To verify whether the gene trap strategy disrupted Dab2IP expression, we examined Dab2IP protein and transcript levels by immunoblot and q-RT-PCR, respectively (Fig. 5D, 5E). At least three major isoforms of Dab2IP protein, ranging from 110–140 kDa, were detected in brain homogenates using our Dab2IP specific antisera. Importantly, only two of the Dab2IP isoforms were knocked down by the gene trap strategy. We investigated the relative levels of Dab2IP transcripts containing PH and GRD domains by q-RT-PCR using specific PCR primers and probes that target these exons (Fig. 5B, 5E). We found that the PH-domain containing transcript was reduced by 99.9% in Dab2IP KO compared to WT littermates, whereas the GRD domain containing transcript(s) was knocked down by 80.9%. Taken together, these results suggest that there may be an alternative *Dab2IP* promoter that is not affected by the gene-trap cassette.

To further investigate if the gene trap strategy selectively affected Dab2IP expression in specific cell types, we performed immunohistochemical analysis (Fig. 5F). Interestingly, Dab2IP expression was abolished in the mossy fiber terminals as well as in the fine punctuate structures within the molecular layer (Fig. 5F). In contrast, modest expression of Dab2IP was detected in PC soma and proximal dendrites as well as in interneurons in the molecular layer (Fig. 5F5). These immunohistochemical results are consistent with the immunoblot and q-RT-PCR results which indicated that some residual expression of Dab2IP remains in the KD mice. In addition, these results suggest that PCs specifically express the high molecular weight isoform of Dab2IP observed in immunoblots.

**Figure 6:**
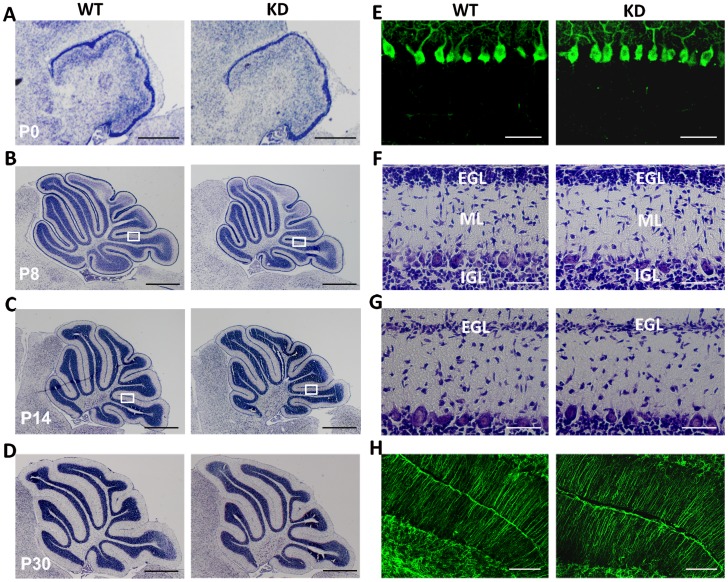
Histological analysis of WT and Dab2IP KD cerebellum. (A–D) Nissl staining of sagittal sections of WT and *Dab2IP* KD cerebella at P0, P8, P14 and P30. (E) Immunostaining of Purkinje cells with anti-calbindin antibodies (green) in WT and *Dab2IP* KD P8 mice. (F–G) Higher magnification views of boxed areas in B and C showing the thickness of the molecular layer at P8 and P14, respectively. (H) Immunostaining of glial fibers using anti-GFAP antibodies in P30 WT and Dab2IP KD cerebella. EGL, external granule layer; ML, molecular layer; IGL, internal granule layer; Scale bars: 500 µm (A–D), 100 µm (E–H).

Dab2IP KD mice were fertile and viable beyond 16 months of age and showed no gross motor behavioral abnormalities, such as those observed in *reeler* mice [12], [13], [17], [36]. Gross cerebellar morphology and architecture was examined by Nissl staining at different postnatal days. We found no obvious abnormalities in foliation of the cerebellar between Dab2IP KD and WT littermates (Fig. 6, A–D). Also, at P8 and P14, there were no detectible differences in the size and morphology of external granular layer (EGL) or internal granular layer (IGL) between Dab2IP KD and WT mice (Fig. 6F–G). Calbindin staining of PCs showed that there was no PCs crowding or misalignment in P8 Dab2IP KD cerebella (Fig. 6E). Lastly, GFAP staining showed no apparent difference in glial scaffold organization between Dab2IP KD and WT mice (Fig. 6H). Together, these data suggest that there are no gross abnormalities in the morphology of Dab2IP KD cerebella.

### Dab2IP Deficiency Delays PC Dendritogenesis

**Figure 7:**
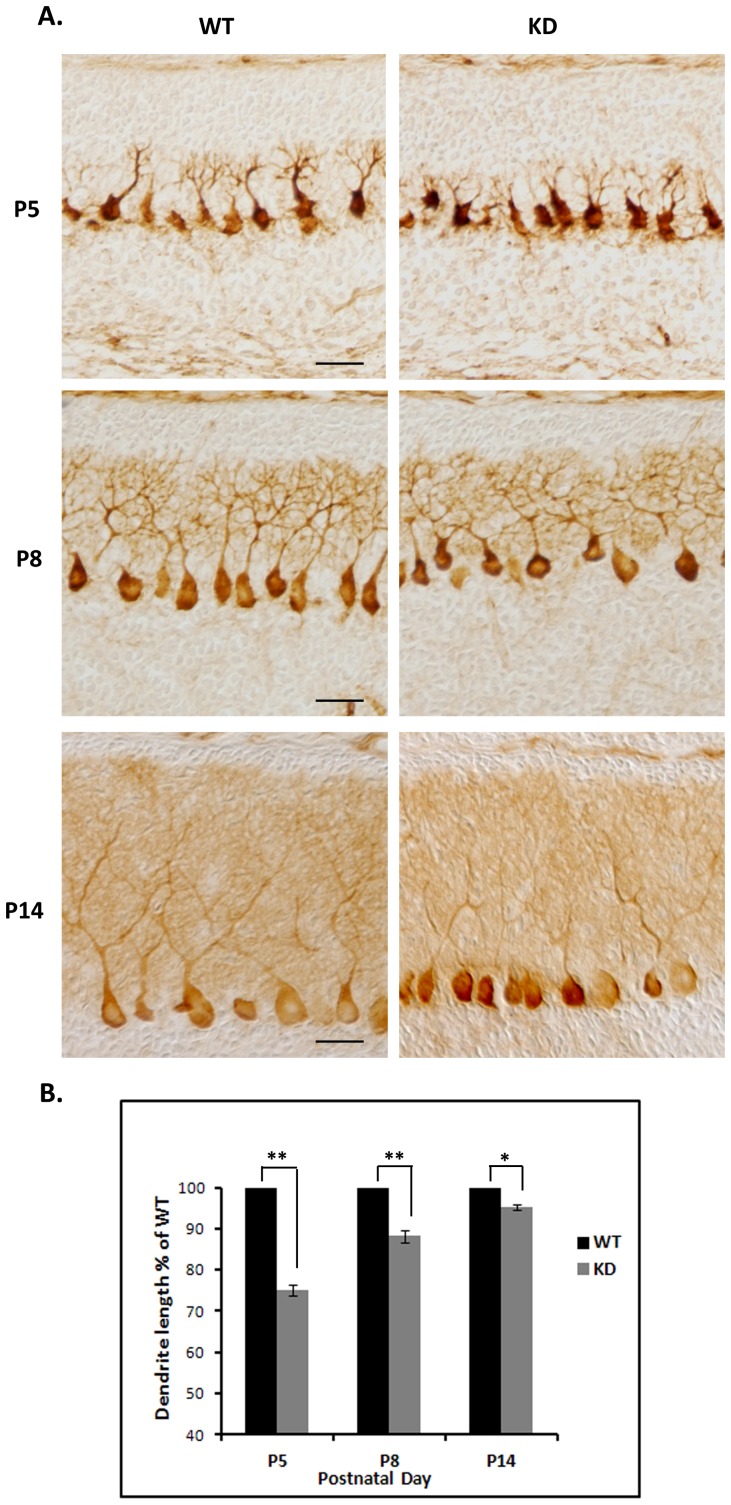
Delayed Purkinje cell dendrite outgrowth in Dab2IP KD mice. (A) Immunohistochemical staining of cerebellar Purkinje cells with anti-calbindin antibody at various postnatal ages (P5, P8 and P14). Scale bar: 50 µm. (B) Measurement of PC dendritic tree length on lobule IV/V in WT (N = 3, 210 observations) and Dab2IP KD (N = 3, 210 observations) littermates at P5, P8 and P14. Values are expressed as means ± S.D. *, p<0.05; **, p<0.01, student’s t-test.

Since Dab2IP is highly expressed in PC soma and dendrites, we investigated if Dab2IP deficiency affects PC dendrite development. Formation of the apical dendrite of Purkinje cells begins soon after PCs complete migration in early postnatal days [37]. We carefully examined the dendritic arborization of PCs labeled with anti-calbindin antibody. At P5, we found that the primary apical dendrite of PCs was stunted and contained numerous processes emanating from the soma in various directions in Dab2IP KD mice compared to WT mice (Fig. 7A). The primary PC dendrite was less pronounced and the overall length of the dendrites was shorter in Dab2IP KD mice compared to WT littermates (Fig. 7A). Quantitative analysis across multiple animals revealed that the length of the PC dendrites were significantly shorter in Dab2IP KD dendrites compared with WT controls (Fig. 7B). This difference was more pronounced at P5 (25% reduction in length, P<0.01) compared to P8 (12% reduction in length, P<0.01) and P14 (5% reduction in length, P<0.05). Interestingly, the length of the PC dendrites in adult Dab2IP KD animals was very similar to WT animals (data not shown). These results indicate that Dab2IP is required for early stages of PC dendrite development.

### Dab2IP Deficiency Affects Parallel Fiber and Climbing Fiber Synaptic Markers

PC dendrite maturation tightly influences the development of PFs and CFs, which form excitatory synaptic contacts with PC dendrites [38]. Early during cerebellar development, PCs are innervated by multiple CFs in the proximal dendrites, which are then eliminated as the synaptic connection between a single CF is strengthened with its PC target [39]. CF elimination is accompanied by the formation of PF synapses on the distal dendrites of PCs [40], [41].

**Figure 8:**
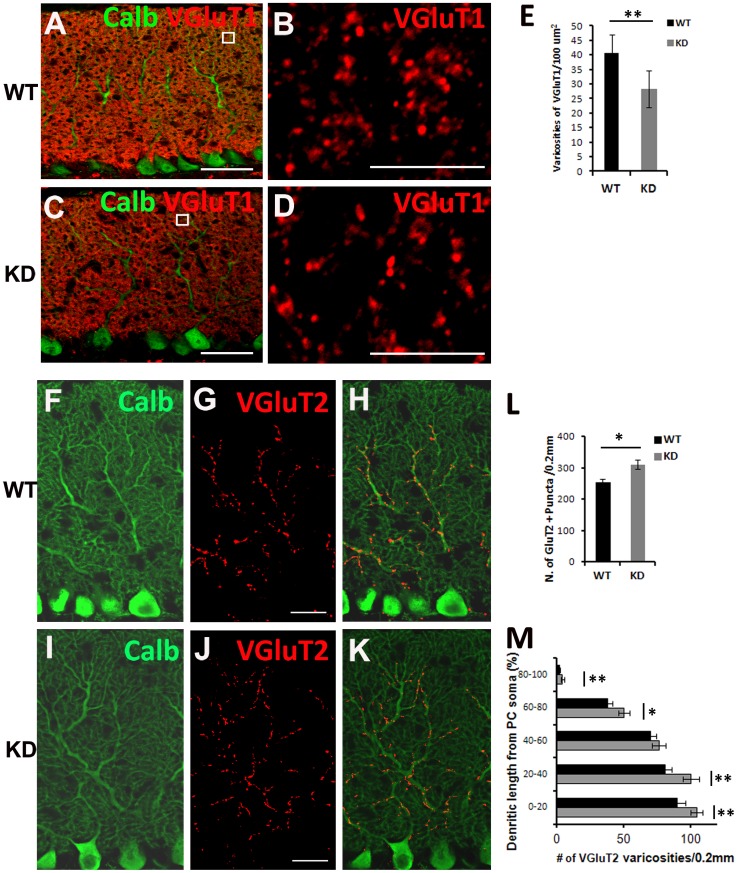
Changes in parallel fiber and climbing fiber synaptic markers in Dab2IP KD mice. (A–D) Confocal images of VGluT1-labeled parallel fibers terminals (red) on PCs stained for Calbindin (green) in P30 control mice (A) or (C) Dab2IP KD littermates. B and D correspond to boxed areas in A and C. Single plane confocal images were used to determine the number of VGluT1 positive varicosities per 100 µm^2^. (E) Quantitation of VGluT1 positive puncta in WT (N = 3, 360 observations) and Dab2IP KD (N = 3, 360 observations) littermates. **, p<0.01, student’s t-test. Scale bars: 50 µm (A, C), 5 µm (B, D). (F–K) Confocal images of VGluT2-labeled climbing fibers terminals (red) on PCs stained for Calbindin (green) in P30 WT (F–H) and Dab2IP KD littermates (I–K). Scale bars: 20 µm. (G, J) Quantitation of VGluT2 positive puncta in WT (N = 3, 171 observations) and Dab2IP KD (N = 3, 195 observations) along the entire length of the molecular layer (L) or in five equal segments from PC soma to the most distal part of the molecular layer (M). **, p<0.01, *, P<0.05, student’s t-test.

As shown above, Dab2IP is expressed in both PF and CF synaptic varicosities labeled with anti-VGluT1 and -VGluT2 antibodies, respectively. Therefore, we performed quantitative analysis of VGluT1 and VGluT2 staining in P30 Dab2IP KD mice compared with WT littermates (Fig. 8). We found that VGluT1-positive PF varicosities were distributed the entire molecular layer in both WT and Dab2IP KD cerebella (Fig. 8A–D). Quantitative analysis showed that the density (number of puncta per unit area) of VGluT1 varicosities were significantly (p<0.01) lower in Dab2IP KD animals (29.21±3.78 per 100 µm^2^) compared to WT littermates (40.40±3.19 per 100 µm^2^) (Fig. 8E). In contrast, we found that the number of VGluT2 positive puncta was significantly (p<0.05) higher in Dab2IP KD mice (310±14.5 puncta per 0.2 mm) compared with the WT littermates (253.7±11.4 puncta per 0.2 mm) (Fig. 8L). Furthermore, significantly more VGluT2 positive puncta were found near the pial surface and on distal branches of PC in the Dab2IP KD mice compared with WT animals (Fig. 8M). These results suggest that a decrease in the number of PF synapses in Dab2IP KD mice is accompanied by an increase in the number of CF synapses on PCs.

The interplay between PF and CF synapses on PC is well-studied using a number of different mouse models [41], [42]. Early during postnatal cerebellar development, a single PC is innervated by multiple CFs. By the second postnatal week, the surplus CF synapses on PCs are eliminated, resulting in a single strong CF innervation. This CF synapse elimination is dependent on PF synaptic activity. Mutant mice lacking granule cells or GluRδ2, which is expressed exclusively in PC dendritic spines that form synaptic contacts with PFs, show defective CF elimination [42], [43]. Thus it is likely that the increase in the CF synaptic marker VGluT2 that we observe in Dab2IP KD animals is caused by the lower number of PF synaptic contacts labeled with VGluT1. This suggests that the lower number of PF synapses in Dab2IP KD mice may be caused by the delay in PC dendritogenesis.

The molecular mechanism by which Dab2IP affects PC dendrite maturation or PF and CF synapse formation in the cerebellum is not clear. Dab2IP has been shown to stimulate Ras GTPase activity in multiple systems [21]. In addition, we found that Dab2IP also exhibits Rap1 GAP activity in cultured cells (unpublished observations). Both Ras and Rap1 GTPases play important roles in axon elongation, branching and synapse formation [44]. In addition, plexin-B1 mediated Ras GAP activity has recently been linked to remodeling of the actin cytoskeleton and dendrite development [45]. Regulation of the cytoskeleton by Ras and Rap1 can be mediated in part through Rho GTPase signaling. Studies in both vertebrate [46] and invertebrate model systems have shown that activation of Rho negatively impacts dendritic growth [47]–[50]. Thus it will be critical to investigate the precise role of Dab2IP in various GTPase signaling pathways and neuronal processes in future experiments.

We have shown previously that Dab2IP directly interacts with Dab1 [18], a cytosolic adapter protein which plays a critical role in Reelin signaling pathway. Reelin controls neuronal migration [4], dendritic development [1], [2] and synaptic plasticity [3], in part through PI3Kinase [8], [10] and CrkL/C3G/Rap1 signaling pathways [15], [51], [52]. Others have shown that Dab2IP regulates PI3Kinase signaling pathway through a direct interaction with the p85 regulatory subunit [26]. We posit that Dab2IP may be a downstream regulator of Reelin signaling and participate in mediating some of the effects of Reelin on dendrite maturation and synaptic plasticity. Defects in dendrite and spine morphology and a reduction in synapse number are observed in *reeler* mice as well as in a number of neuropsychiatric disorders [53], [54]. It would be interesting to investigate if Dab2IP mediates some of the cellular effects of Reelin and whether it could play a role in any of the Reelin associated neuropsychiatric disorders.

## Supporting Information

Figure S1 **Quantitation of VGluT1-positive varicosities in molecular layer of cerebellum.** (A) Three images (black boxes) were collected from cerebellar lobule IV/V on 10 mid-sagittal sections from 3 independent WT and Dab2IP KD animals. (B) Single plane confocal image (189×magnification) was divided into four equal quadrants and the number of VGluT1-positive varicosities were counted in each quadrant. (C) Example of manual puncta delineation on a few varicosities from the lower left quadrant in B.
(TIF)
