# Supplementary figures and images for: Dab2IP GTPase Activating Protein Regulates Dendrite Development and Synapse Number in Cerebellum
Source: PLoS One. 2013 Jan 9;8(1):e53635. doi: 10.1371/journal.pone.0053635 (PMC3541190; doi:10.1371/journal.pone.0053635)

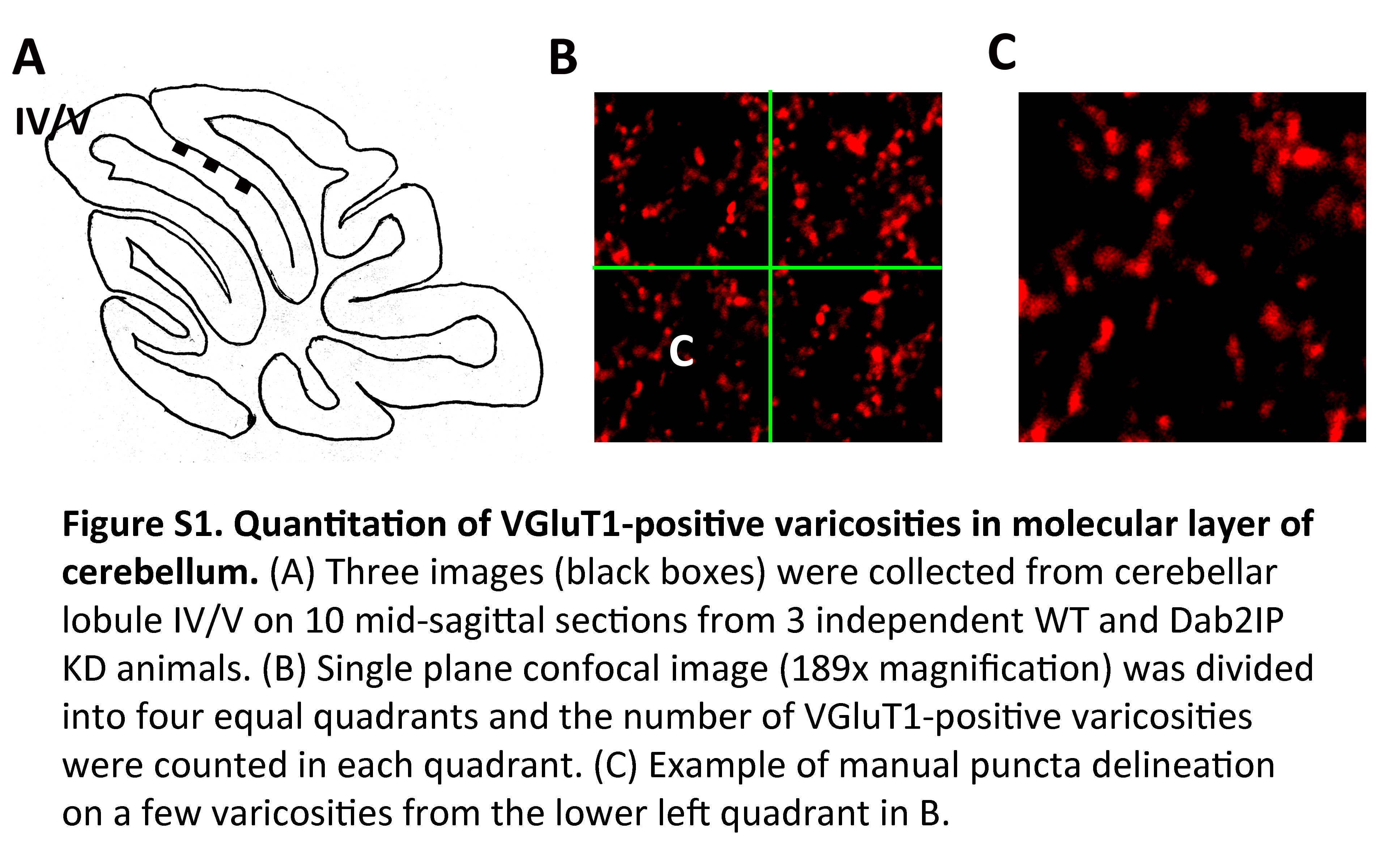

Supplement: Figure S1 — Quantitation of VGluT1-positive varicosities in molecular layer of cerebellum. (A) Three images (black boxes) were collected from cerebellar lobule IV/V on 10 mid-sagittal sections from 3 independent WT and Dab2IP KD animals. (B) Single plane confocal image (189×magnification) was divided into four equal quadrants and the number of VGluT1-positive varicosities were counted in each quadrant. (C) Example of manual puncta delineation on a few varicosities from the lower left quadrant in B. (TIF) [file pone.0053635.s001.tif]
